# The Impact of Extreme Temperature Shocks on the Health Status of the Elderly in China

**DOI:** 10.3390/ijerph192315729

**Published:** 2022-11-25

**Authors:** Yanran Chen, Xuezheng Qin

**Affiliations:** 1 School of Economics, Capital University of Economics and Business, Beijing 100070, China; 2 School of Economics, Peking University, Beijing 100871, China

**Keywords:** climate change, extreme temperature, population health, aging

## Abstract

With the accelerating process of climate change, long-term exposure to extreme temperatures could threaten individuals’ physical health, especially for the vulnerable population. This paper aims to investigate the long-term effects of extreme temperature exposure on the health of the elderly in the context of climate change and aging. Different from most of the existing literature in environmental economics, we define the relative extreme temperature exposure based on the local temperature pattern. By combining a large national household survey and nationwide meteorologic historical data, this study provides empirical evidence that heat exposure days and cold exposure days during the past year both significantly affect the physical health of middle-aged and elderly groups, controlling for city, year, and individual fixed effects. The effect on individual physical health has certain seasonal characteristics and is heterogeneous across populations. Additionally, cooling and heating equipment are effective in alleviating the reverse impact of heat and cold exposure. The estimation is robust and consistent across a variety of temperature measurements and model modifications. Our findings provide evidence of the long-term and accumulative cost of extreme temperature to middle-aged and elderly human capital, contributing to helping the public to better understand the full impact of climate change.

## 1. Introduction

Climate change has become one of the greatest challenges to humanity since the 21st century. The relationship between the economic well-being of human societies and the unpredictable and evolving process of climate change has become a particularly critical issue in climate change economics [1]. Based on the theoretical foundation of health human capital and climate change economics, there is a growing study in the fields of economics and public health that has illustrated that its continuous development has posed a great threat to the health of the population in many ways [2,3]. At present, the magnitude of global warming has exceeded 1.2 °C compared with the average level before industrialization in the world, resulting in profound, long-term, and continuously deteriorating health effects [4,5]. Since the process of industrialization and urbanization, the world’s economies have experienced unprecedented high-speed development. Aside from the global climate change, urban heat island effects exacerbate the impacts on local climate change, by increased exploitation and utilization of natural environmental resources, as well as social and economic activities.

In addition to the global warming trend, another important feature of climate change is that the intensity and frequency of extreme temperature events are also increasing [6]. According to the latest data, the total number of global heat wave exposures reached 220 million in 2018, breaking the highest value since the record was established in 2015 [7]. The average number of days of exposure per person per year increased by 1.4 days from 2000 to 2017, compared to baseline data for 1986–2005 [7]. In 2019, it is emphasized, for the first time, that the significant impact of temperature on health, and even small changes in temperature can pose a significant threat to human health [7,8,9], such as the increase in infectious diseases [8,10], affecting the reproductive quality of newborns and further affecting human mortality and fertility rates [11,12].

Early research on the threats and challenges of extreme temperatures on health human capital also focused on the effects on individual physical health. The medical and public health fields first began to focus on the strong link between temperature and health, with studies showing that as the temperature exceeds the tolerance range of an individual’s body (generally thought to be >30 °C), it will begin to affect the function and behavior of the body [13]. In addition, the available evidence also suggests that high-temperature stress and heatstroke will lead to the rapid deterioration of cardiovascular and respiratory health, which will greatly increase morbidity and mortality [14,15] and furthermore, to increased morbidity and mortality from other diseases [16]. The severe heat wave exposure in Europe in 2003 caused about 50,000 deaths and caused a rapid increase in the incidence of lung diseases and cardiovascular diseases of all ages, among which the threat to the health of the elderly is the most serious [17,18]. Many scholars also have shown that high temperatures and drought will directly increase the risk of suicide and increase the mortality rate of the population [19,20,21,22]. In addition, data from different countries explore the relationship between temperature rise and hospital visits. High temperatures have been reported to increase the number of emergency hospital visits in nations including Australia [23], Canada [24], and the United States [25].

On the other hand, extremely cold weather will also have a great impact on health. Acute large-scale cold wave events often lead to large-scale influenza, entailing risks to human health [26]. However, compared with extreme heat, the existing literature is limited to the discussion of the health threats of extreme cold temperature exposure.

With the acceleration of global climate change and the aging process, the elderly is one of the groups that are most vulnerable to negative impacts. The latest annual report released by the Lancet (2020) further emphasizes that the elderly is one of the most vulnerable groups affected by extreme temperatures. Since 2010, compared with the baseline from 1986 to 2005, the number of days of extreme high-temperature exposure of people over 65 years old has significantly increased. In particular, in 2019, the number of extreme high-temperature exposures of people over 65 years old reached 475 million, which was nearly 160 million more than the highest record set in 2016 [6]. From 2000 to 2018, the death rate related to extreme high temperatures of those 65 years old increased by 53.7% worldwide, and the death toll reached 296,000 in 2018 [27]. As older people have fewer coping mechanisms to adapt to climatic conditions physically and mentally [28,29,30], greater psychological susceptibility and a lack of social resources mainly lead to their vulnerability. As a result, they are more likely to suffer from the negative effects of climate change [31], and have a significantly higher risk of death during extreme weather events [32]. The interaction between the adverse effects of climate change and the characteristics of older persons poses great challenges to health and environmental justice. Additionally, the vulnerability of the elderly in China needs greater attention.

China became an aging society in 1999 and now has 137.6 million elderly people, ranking first in the world [33]. However, against the background of climate change, the vulnerability of the elderly in China has not been paid sufficient attention by the public. Some research has shown that the risk of suicide among the older population is significantly higher than in other age groups [34] and four to five times the average risk of suicide in China [35]. Although the elderly population represents only 8.9% of the Chinese population, 39.2% of suicide deaths occur in these groups [33]. According to the latest data, the number of deaths related to extreme temperatures over 65 years old in China reached 62,000 in 2018, ranking first in the world [6]. Therefore, identifying health risk factors of extreme temperatures for the elderly will play a vital role in elucidating climate risks, improving the further interventions and corresponding adaptation behaviors, and protecting the health human capital and the welfare of the elderly population.

By reviewing the current empirical economic research on extreme temperatures on individual health, we find the following: Firstly, most studies focus on the negative effects of extreme high-temperature exposure on health, while few studies include the effects of extreme low-temperature exposure [26]. Secondly, most studies discuss more serious physical health indicators (such as mortality and suicide). However, the cumulative impact of extreme temperature will also cause a slow deterioration of individual health status, chronic disease management and daily activities. Some non-acute physical health indicators are also worthy of attention. Thirdly, most studies use macro-level, administrative data, which cannot be used to analyze the behavior characteristics of individuals in depth. Finally, in a few studies using micro-survey data, the self-reported health level of individuals is often used to measure the health status of individuals, and there is a lack of more objective and comprehensive health indicators for analysis and discussion.

In order to fill these gaps, this paper will try to identify the cumulative relationship between long-term extreme temperature exposure and the physical health of middle-aged and elderly people. Firstly, this paper establishes a fixed effect model of three-year unbalanced panel data based on the large-scale micro-household survey data nationwide. We try to identify and estimate the long-term, local, and cumulative effects of slowly changing temperature conditions in specific areas on individual health status, especially on vulnerable elderly groups through empirical econometric models. Secondly, this paper further explores the seasonal characteristics of extreme temperatures on physical health status and the heterogeneity of these effects among different populations, mainly including age, gender, and residential areas. Thirdly, this paper analyzes the impact of air conditioning and various types of heating equipment on individual adaptability in the process of coping with extreme temperatures. Finally, this paper carries out abundant robustness tests on the empirical model in the aspects of sample selection, index construction, and model selection.

## 2. Methodology

### 2.1. Data Source

This paper considers panel data from the China Health and Retirement Longitudinal Study (CHARLS) in 2011, 2013, and 2015, which was organized and implemented by the China Social Science Research Center of Peking University, with the purpose of collecting information about the health status of the Chinese population and studying the aging problem. The CHARLS is similar to the national health and pension tracking surveys designed and implemented by other developed countries (such as HRS in the United States, SHARE in Europe, EISA in the United Kingdom, KIOSA in South Korea, and ASTAR in Japan), aimed at collecting basic personal information, information on family structure, health status, physical measurements, medical service utilization and medical insurance, work, retirement and pension, income, consumption, and assets, and basic community information of people aged 45 and above and their spouses [36]. In the sample selection, 150 counties were selected using the scale sampling method according to probability proportion, 3 villages or communities were then randomly selected in each county, and people over 45 years old were then randomly selected as the main respondents in each village or community. Once selected, their spouses were included as respondents, regardless of how old they were (For details and introductions on the dataset, please refer to the official website: http://charls.pku.edu.cn/, accessed on 19 November 2022).

The meteorological data in this paper come from the Climate Data Online (CDO) of the U.S. National Oceanic and Atmospheric Administration (NOAA). CDO provides free archives of global historical weather and climate data (including data on China) to the public. These data include daily, monthly, seasonal, and annual temperature, precipitation, wind, and radar data (CDO provides free public access to the global historical weather and climate data, including China. For details and introductions of the dataset, please refer to the website: https://www.ncdc.noaa.gov/cdo-web/, accessed on 19 November 2022). The original China data come from the real-time and historical meteorological data of national reference meteorological stations collected and released by the China Meteorological Administration (This raw data is now available directly from the National Weather Science Data Center; see the official website http://data.cma.cn/, accessed on 19 November 2022).

According to geographical latitude and longitude information, each weather station was classified as a city (if there are multiple weather stations in a city, the meteorological data were averaged). If the city where the respondent is located was covered by the meteorological database, the meteorological data of the city were used. If the city was not covered by the meteorological database, referring to the literature, the meteorological data came from the weighted average of the data of observation stations within 100 km of the city center, where the weight is the reciprocal of the distance from the monitoring station to the city center [37,38]. Based on the matched meteorological data and the information of the sample city provided in the CHARLS database, the above meteorological data were matched with micro-household data, so that the exposure days of extreme temperature events at a specific time can be calculated for each individual.

### 2.2. Index Construction

#### 2.2.1. Extreme Temperature

In the basic model, this paper quantifies “extreme temperature exposure” as the number of days (0–365 days) that respondents were exposed to extreme weather in the past year (i.e., 2010, 2012, and 2014).

The majority of the available literature address extreme temperature issues by adopting absolute temperature values as the measurement. Climatology literature first develop the relative index of measuring the deviation from the historical values when monitoring the severity of droughts or precipitation [39]. Referring to the similar practice of Andalon et al. (2016) when investigating the effect of extreme temperature shocks on infant birth weight based, if the daily average temperature on a given day is higher or lower than the monthly average temperature in local historical precedents by 1.96 standard deviations (that is, it shows that there is a significant difference between the daily temperature and the monthly average temperature in the same period of history), such a day is defined as local extreme temperature day. Then, the cumulative exposure days of the weather station in a year were calculated, so as to obtain the exposure level. In addition, local historical precedents in the past 30 years from 1980 to 2010 of the weather station were selected as references to calculate the daily extreme temperature exposure.

Specifically, based on the historical meteorological data from 1980 to 2010, the average monthly temperature and its standardized deviation were calculated as the local historical reference temperature for each city. The daily average temperature was then compared with the local historical monthly average temperature in the same period, and the Z-Score was calculated. In the following formula, subscript *m* represents a specific month and *d* represents a specific date. The Z-score on day *d* of month *m* is equal to the difference between the average temperature of that day and the historical average temperature of month *m*, which is then divided by the standard deviation of the temperature of month *m*, so as to obtain the relative deviation degree of the temperature of that day. Therefore, the Z-Score measures how many standard deviations the daily temperature deviates from the historical temperature in the same period. If the average temperature on that day deviates from the average monthly temperature in the same period of history by more than 1.96 SD, that day is defined as a “relative extreme temperature exposure day”. If the deviation is positive, it is defined as an extreme high-temperature exposure day; otherwise, it is defined as an extreme low-temperature exposure day. Finally, this paper defines the cumulative exposure days in the year before the interview as the exposure level of each individual. In this paper, it is referred to as the average “local extreme high temperature exposure (days)” or “local extreme low temperature exposure (days)” of individuals.

(1) $$Z-Score_{md}=\frac{Temp_{md}-Mean_{m}}{SD_{m}}$$

#### 2.2.2. Physical Health

This paper measures the individual’s physical health from multiple dimensions. In addition to the self-reported health level commonly used in the literature (the discrete value is 1–5; the larger the value, the higher the self-reported health level), considering that the main respondents are middle-aged and elderly people and the self-reported health level has a certain subjectivity, two variables commonly used in labor economics and health economics are also employed, namely, “ADL/IADL” and “suffering from chronic diseases”, to measure the true physical health level and disease status of individuals. In the CHARLS questionnaire, interviewees were asked whether they could independently complete 6 daily activities (ADL) and 5 functional daily activities (IADL). The ADL/IADL index would be a dummy variable, and it takes the value of 1 if any one of the activities cannot be completed independently, and 0 otherwise. Similarly, the interviewer also asked about the prevalence of 14 common chronic diseases, and the number of chronic diseases suffered by individuals was calculated (the value is 0–14; the higher the value, the more chronic diseases suffered).

In addition, considering that the samples are mainly middle-aged and elderly people, this paper applies the individual cognitive ability as a proxy variable of the individual aging process and discusses individual health from another dimension (Cognitive ability has also been used as one of the measures of mental health in the literature. While considering the middle-aged and elderly sample and that the cognitive abilities measured in this questionnaire mainly involve mathematical ability and memory, it is regarded as a measure of aging in middle-aged and elderly individuals and as a part of physical health in this paper). The CHARLS questionnaire was used to test the individual’s word recall tests and mathematical calculation ability, which was used to measure the individual’s cognitive ability. Specifically, referring to the practices in related literature, two levels of word recall tests (including long-term memory and short-term memory) and mathematical calculation tests were used to measure the cognitive ability of middle-aged and elderly individuals [40,41], and they are often used to determine the aging process and health status of individuals. In the word test, the interviewee read 10 words in turn, and the interviewee was asked to recall these words immediately. The number of correct words recalled was the score of individual short-term memory. After a period of time, the interviewee was asked to recall the repeated words again, and the number of correct words recalled was regarded as the score of individual long-term memory. Therefore, the scores of short-term memory and long-term memory were between 0 and 10. In the mathematics test part, respondents were asked to answer five math calculation questions (that is, answer 100 questions five times in a row minus 7 answers), and the number of questions answered correctly was regarded as the individual mathematics test score. Therefore, the mathematics ability score was between 0 and 5.

#### 2.2.3. Descriptive Analysis

Table 1 reports the descriptive statistical results of the database used in this paper. Panel A reports the descriptive results of different dimensions of physical health indicators, namely, self-reported health status, ADL/IADL, chronic diseases, and three cognitive ability variables (short-term memory, long-term memory, and mathematics test scores). From the results of descriptive statistics, the average self-rated health status of the samples was 3, which represents a “general” physical health state. On average, there were 27% of the sample could not carry out at least one of the daily activities independently, and each person suffered from 1.19 chronic diseases on average. The average scores on short-term memory, long-term memory, and mathematics tests were 3.58, 2.85, and 2.63, respectively.

Panel B shows the measurement of extreme temperature exposures in detail. Generally speaking, after matching the meteorological database with the CHARLS database, there were about 100 days of extreme high-temperature exposure and 77 days of extreme low-temperature exposure every year in the samples selected in this paper based on the 1.96 cutoff of Z-scores.

Panel C presents the basic descriptive statistics of the control variables in the model, including common meteorological variables (including wind speed, sunshine time, relative humidity, air pressure, and PM2.5) and demographic variables (including age and its square term, education level, marital status, participation in endowment insurance, participation in medical insurance, the number of children, the number of sons, and per capita annual expenditure).

### 2.3. Empirical Design

In order to identify the impacts of extreme temperature exposure on the health of middle-aged and elderly people, this paper establishes a panel data fixed effect model for quantitative empirical analysis, which has been widely used in climate economics literature [42,43]. The fixed effect model exploits the advantages of panel data with the least square dummy variable (LSDV) estimation method, thus eliminating the endogenous problems caused by time-invariant variables (such as gender, marital status, etc.), and controlling the two-way fixed effects at individual and time levels. Based on the unbalanced panel data of the CHARLS in 2011, 2013, and 2015, this paper establishes a fixed effect model with the following formula, and controls the fixed effects at the individual, time, and city levels.

(2) $${\mathrm{Physical}\;\mathrm{Health}}_{\mathrm{it}\;}{=\alpha }_{0\;}{+\beta }_{1}{\mathrm{Exposure}}_{\mathrm{it}}{+\beta }_{2}W_{\mathrm{it}}{+\beta }_{3}X_{\mathrm{it}}{+\gamma }_{i}{+\eta }_{c}{+\mu }_{t}{+\varepsilon }_{\mathrm{it}}$$

where the subscript *i* represents the individual code, *t* represents the year code, and *c* represents the city code. ${\mathrm{Physical}\;\mathrm{Health}}_{\mathrm{it}\;}$ denotes physical health indicators. ${\mathrm{Exposure}}_{\mathrm{it}}$ indicates the number of days an individual has been exposed to extreme temperatures in the past year; $W_{\mathrm{it}}\;$ indicates other environmental variables that may affect individual health, such as precipitation, wind speed, air pressure, humidity, sunshine duration, and air pollution; $X_{\mathrm{it}}$ represents other demographic control variables; $\eta_{c}$ is the fixed effect of the city level; $\gamma_{i}$ is an individual fixed effect that does not change with time and will be eliminated in the estimation of the panel data fixed effect model. In the environmental economics field, recent literature has examined those other weather-related risks, such as serve air pollution [37,38,44,45,46] and the impacts of sunshine or wind [47,48], which may also show a significant influence on health human capital. In order to further consider the influence of other meteorological factors on individual health, abundant meteorological variables, including precipitation, wind speed, sunshine duration, relative humidity, air pressure, and PM2.5, are considered and included as control variables.

The coefficient of interest is $\beta_{1}$, which represents the average change of physical health outcome variables of middle-aged and elderly individuals when the number of days exposed to extreme temperatures in a year increase by one day, while other influencing factors remain unchanged. The key identification hypothesis of this empirical strategy is that, under the condition of continuous changes of multiple outcome variables of individual physical health, the change in temperature has nothing to do with other unobserved determinants of physical health for a given individual. Temperature realizations are as good as random after accounting for spatial and temporal fixed effects [22]. On the one hand, against the background of climate change, the occurrence of weather events such as extreme temperatures are largely unpredictable in advance, especially in the process of climate change [11,49]. On the other hand, by constructing an index of local extreme temperatures, local variations in temperature are rendered more exogenous than absolute temperature values, as local temperature deviations are portrayed by subtracting historical contemporaneous temperatures, controlling for individual expectations of the environment in which they live, and considering their adaptation to long-term living conditions. In addition, the panel data fixed effect model accounts for the mean deviation of variables in the estimation process, thus eliminating the endogenous problems caused by time-invariant variables, and controlling the fixed effects at individual and time levels, thus controlling the endogenous problems to a certain extent.

It is worth noting that, with the acceleration of climate change and the frequent occurrence of extreme weather events, more technologies and equipment can help people better adapt to extreme temperatures. Based on this, this paper also discusses the difference in the influence of extreme temperature exposure on individual health when individuals have other cooling and heating equipment, such as air conditioners. Therefore, this paper further characterizes the seasonal characteristics of extreme temperature exposure by calculating the local extreme high-temperature exposure days and local extreme low-temperature exposure days in summer (June to August) and winter (December to February of the following year). Based on this measurement, the heterogeneous effects of extreme temperature exposure on the health status of people with air conditioning or heating equipment were further discussed.

Furthermore, in order to verify the robustness and consistency of the model, rich robustness tests on index construction, model setting, and identification methods in the benchmark model and the extended model are also carried out. This paper mainly focuses on the multi-dimensional verification and design of the measurement standard of “extreme temperature exposure”, which is also discussed and compared in the *Robustness Checks* section.

## 3. Results

### 3.1. The Basic Model

Table 2 reports the regression results of the basic model, which uses the deviation of the daily mean temperature from the historical mean temperature in the same period as the definition of extreme temperature. Column (1) is listed as the individual’s self-reported health status (the individual self-reported health statuses are discrete categorical variables, taking values of 1–5, but considering the difficulty of the estimation of fixed-effects models, it is hard for discrete categorical variables to maintain a high degree of variation even after the mean deviation calculation when fitting the non-linear setting. In order to avoid identification problems caused by perfect collinearity. In this paper, they are treated as continuous variables and estimated using a linear panel data LSDV model), (2) is shown as the ADL/IADL (both ADL/IADL and chronic diseases were also treated as continuous variables for the same reasons as above), (3) is presented as the individual’s chronic disease, and (4)–(6) are the results of short-term memory, long-term memory, and the mathematics test, respectively. Regression results indicate that both extreme high-temperature exposures and extreme low-temperature exposures have significant adverse impacts on individual health.

Specifically, when the middle-aged and elderly populations are exposed to extreme heat exposure for an extra one day, their self-reported health level decreased by 0.0016, daily activities disorders increased by 0.0013, and the number of chronic diseases increased by 0.0018. According to the descriptive statistics listed in Table 1, extreme heat exposure for an average of 100.77 days per year would deteriorate the self-reported health of middle-aged and elderly individuals in the sample by 3.22% (Referring to the calculation method commonly used in the field of public health, the impact value = 0.0016 units × average annual number of days of heat exposure 100.77/total self-assessed health rating 5 = 3.22%, and the following calculation method is the same), and their ADL/IADL and chronic diseases increased by 1.19% and 1.30%, respectively. On the other hand, extreme cold exposure also caused a similar amount of damage to physical health. However, for the self-reported health status and the number of chronic diseases, the impact of extreme heat exposure was about twice larger than that of extreme cold exposure. To sum up, based on the direction and significance of the coefficients, extreme heat exposure and extreme cold exposure had a significant negative impact on self-reported health status, daily activity disorder, and chronic disease of middle-aged and elderly people. When compared with extreme cold, populations are more sensitive to extreme heat exposures.

Similarly, from the perspective of cognitive ability, both extreme heat and cold exposures had obvious negative effects. In detail, when exposed to extreme heat temperature for an extra one day, the scores of long-term memory and the mathematics test decreased by about 0.0015 and 0.0016 points, respectively. Annually, the scores of long-term memory and mathematics test would decrease by 0.15 and 0.16 points, and the score level decreased by about 1.51% and 3.22% (similarly calculated according to the average annual exposure of 100.77 days in the sample). Moreover, exposure to cold temperatures significantly endangered the memory and mathematical calculation ability of middle-aged and elderly individuals. While extreme cold exposure had a significant negative impact on memory (including long-term memory and short-term memory), and the impact was higher than that of extreme heat, but had no significant impact on individual mathematical ability.

The above results show that long-term exposure to local extreme temperatures, high or low, had a significant negative impact on the health status of middle-aged and elderly people in different dimensions. The regression results suggest that both extreme high-temperature exposure and low-temperature exposure significantly deteriorate individual self-reported health status, daily activity disorder, and chronic disease. The negative effect of cold exposure on individual memory was much higher than that of heat exposure, and heat exposure also led to a significant decline in individual mathematical ability.

### 3.2. Seasonal Patterns and Adaptations

AC and heating are two main resources that are used to mitigate the effects of uncomfortable temperatures. To further estimate the size of this mitigation effect, we conduct a subsample analysis of the populations with and without AC/heating in summer and winter, respectively. The regression results of the seasonal patterns of extreme temperatures are reported in Table 3, in which Panel A and B show the impacts of extreme heat/cold exposure days in summer/winter of middle-aged and elderly individuals. Panel C and D present whether the prevalence of air conditioners and various types of heating equipment can help alleviate the negative impact of temperature changes on the health status of middle-aged and elderly people.

Panel A and B of Table 3 reflect the seasonal patterns of the impact of extreme temperature on physical health. The results show that the negative effects of extreme heat exposure on health were comprehensive and significant. In summer, compared with cold exposure, extreme heat exposure had a significant negative impact on self-reported health status, ADL/IADL, and chronic diseases, and the magnitude was greater compared to cold exposure. In addition, the impact of extreme cold exposure days on individual self-reported health was highly similar to that of extreme heat, and significant negative effects were present. However, the harm of extreme heat on cognitive ability was not significant, and extreme cold exposure had a significant negative impact on individual long-term memory scores. In winter, extreme high temperatures also led to different dimensions of damage to individual health, and the impact was higher than that of basic regression. Comparatively speaking, the negative impact of extreme cold exposure in winter was not significant.

That is to say, the detrimental effects of extreme heat on physical health are significant and comprehensive, which cannot be neglected both in summer and winter. For summer, the extreme heat of a “hotter summer” had a significant negative impact on individual health. The main reason for this may be that the climate in summer in China is characterized by a generally high temperature. When extreme high-temperature events occur on this basis, according to the definition of this paper, the average daily temperature needs to be higher than the historical temperature in the same period by more than 1.96 SD, which means that the absolute temperature on that day is already quite high. The progress of modern industrialization and the urban heat island effect further amplify the negative effects of extreme heat. If outdoor workers or indoor workers with no temperature-regulating equipment, such as air conditioner, extreme high temperatures pose a great threat to the tolerance of individuals, thus causing significant deterioration in individual health. Regarding winter, a “hotter winter” is an abnormal temperature in the corresponding season has a greater effect on an individual’s health. The reason for this may be that there is a large temperature gap in different areas of China in winter. Individuals living in extremely cold areas tend to reduce their daily activities and economic production activities in the winter, and generally alleviate discomfort caused by extreme cold with heating equipment. Compared to air conditioning, heating equipment in China is very prevalent in both rural and urban areas. It is worth noting that in a lot of heating equipment it is often difficult to adjust the setting temperature promptly once it starts. Abnormal extreme high temperatures in winter often do not satisfy general expectations, and short-term adaptation mechanisms are lacking. A cold environment in winter leads to a high incidence period of various cardiovascular and cerebrovascular diseases and lung diseases, so sudden abnormal extreme temperatures in winter often have a great impact on the health status of vulnerable groups such as middle-aged and elderly people. Therefore, the conclusion of the basic regression is further verified and enriched, and extreme heat exposure has more comprehensive and significant impacts across seasons.

On the other hand, air conditioning and various heating equipment (including electricity and the use of coal, oil, or gas) have been recognized as tools that help people adapt to environmental temperature changes. In order to further estimate whether air conditioning and heating can help alleviate the overall impact of temperature changes on the health of middle-aged and elderly people, this section compares people with air conditioning and any kind of heating equipment or not when extreme temperature exposure events occur. The estimated results are shown in Panel C and D of Table 3.

The estimated results are basically in line with expectations and are consistent with conclusions in the existing literature [22]. If there is air conditioning in the household, heat exposure in summer will not significantly affect an individual’s self-reported health status, daily activities (ADL/IADL), or chronic diseases and can significantly improve the individual’s long-term memory, short-term memory, and mathematics test scores. While in the absence of air conditioning, compared with the basic regression results, the impact of heat exposure in summer on the health of middle-aged and elderly people is still significant. In addition, different types of heating equipment also show that reducing cold exposure in winter has a comprehensive positive impact on health status. The regression results of Panel D in Table 3 suggest that, if the household has at least one heating source, cold exposure in winter will not have a significant negative impact on the health indicators. However, there is no heating equipment in the household, and a significant negative impact of cold exposure in winter remains.

These results show that air conditioners and various types of heating equipment play an effective role in helping people adapt to changes in ambient temperature. Most previous studies emphasize the positive role of air conditioning in improving individual adaptive behavior against the background of climate change, and few studies have discussed its positive role in alleviating extreme cold exposure in winter using household heating equipment information. In our sample, about 63.12% of the households had at least one type of heating equipment, while only 28.06% of the sample households had air conditioning. Due to the lack of air conditioners in households (especially in rural households), extreme heat events in China have a strong and significant impact on the health status of middle-aged and elderly individuals. However, since most homemade heating methods (such as burning coal, firewood, or charcoal fire) of rural residents are less expensive to obtain and utilize, the prevalence of heating equipment in rural areas is higher. The elderly groups tend to have a certain capacity for adaptation and resistance to extreme cold temperature events that occur in winter, thus mitigating the effects of extreme cold temperature exposures. On the other hand, more reports and studies emphasize the negative environmental impacts and potential health hazards caused by the overuse and wide application of various air-conditioning and refrigeration technologies, such as increasing greenhouse gas emissions, electricity load, and urban heat island effects, thus aggravating the process of climate warming in turn. However, the advantages of air-conditioning and some heating equipment in improving the adaptability of individuals and families to the environment against the background of climate change are often ignored.

## 4. Heterogeneity

This section further explores the influence of extreme temperature exposure on the health status of different characteristic groups.

First of all, there are great differences between the middle-aged group and the elderly in terms of physical health, psychological state, the social division of labor, work arrangement, and living habits. On the one hand, based on China’s existing retirement policy, those over 60 years old gradually retire from the labor market, and retirement also leads to great changes in the lives of middle-aged and elderly people, thus leading to physical and mental discomfort [50,51,52]. On the other hand, as age grows, greater psychological susceptibility and a lack of more social resources will aggravate their physical and psychological vulnerability, so it is generally believed that those over 60 years old are often more vulnerable [31]). Secondly, considering the vast territory of China, there are great differences in climate features and living habits between the north and the south, and the differences in extreme temperatures may vary. Thirdly, due to the dual structure of urban and rural areas in China, there are great differences between urban and rural areas in infrastructure, economic level, production and living activities, pension arrangements, and health status [53,54,55]. Finally, previous studies have shown that, compared with men, women are more vulnerable to short-term weather shocks and short-term extreme temperatures [7,56,57], so extreme temperature shocks caused by climate change also present gender inequalities. Based on the above discussion, this section carries out a sample analysis by age, gender, residential area, and urban–rural distribution, and Table 4 reports the results of the sample regression.

Panel A of Table 4 reports the sub-sample results of those over and under 60 years old. The main regression results were very similar to those in the basic regression. From the perspective of age, the increase in extreme heat exposure days both increased the negative impact of self-reported health status and chronic disease prevalence of those over 60 years old and those under 60 years old, and had a greater impact on those over 60 years old. However, the increase in extreme cold days shows less difference across the two groups. The results of Panel A in Table 4 further prove that, in the context of climate change, compared with the middle-aged group, the elderly group over 60 years old is one of the most vulnerable groups due to the many basic diseases, poor adaptability, and accelerated aging process. The results of this section further emphasize the vulnerability of the elderly in developing countries and prove that climate change not only causes the deterioration of the ecological environment, but also challenges the fairness and justice of the social environment.

Panel B of Table 4 shows the sub-sample regression according to the north–south classification of the region (divided according to the north–south boundary of the Qinling Mountains–Huaihe River). The coefficient characteristics and main conclusions of the sub-sample results are similar to those of basic regression. From the perspective of extreme heat exposure, compared with the northern group, the southern group was greatly and significantly negatively affected in terms of self-reported health, chronic disease prevalence, short-term memory, and long-term memory. As for extreme cold exposure, the southern group also suffered a greater negative impact on self-rated health and chronic disease prevalence, but there was little difference in cognitive ability between these two groups. The results of Panel B further reflect the great differences in climate characteristics in the different regions, which also leads to the differences in individual adaptive behaviors.

Panel C of Table 4 presents the urban–rural differences in the basic regression. The results show that both rural and urban residents are affected by local extreme temperature exposure, but the impact is also heterogeneous. Generally speaking, in addition to self-reported health indicators, compared with urban areas, the physical health indicators of the rural population are more susceptible to local extreme temperatures. Local extreme high-temperature exposure has a statistically significant negative impact on self-reported health, ADL/IADL, chronic disease prevalence, long-term memory, short-term memory, and mathematics test scores of rural elderly groups. The inequality between urban and rural groups exposed to extreme cold is lower than that of extreme heat, but the negative impact of rural groups on self-rated health, chronic disease prevalence, long-term memory, and short-term memory is higher than that of urban areas. This further proves that, due to the differences in infrastructure and economic level, urban residents have a higher ability to adapt to local extreme temperature exposure. Since economic activities and social development in rural areas are highly dependent on the stable operation of ecosystems, especially for agricultural production, which are often the sectors most directly affected and most vulnerable to the adverse effects of climate change, and the health status and socio-economic levels of the rural population are also more vulnerable to extreme weather events. In addition, compared with the urban population, the social and economic status of the rural population in China is relatively low, which means that they have fewer social resources and a relatively poor ability to adapt to abnormal natural events. The existing literature focuses more on the disproportionate adverse effects of natural disasters and the short-term weather shocks on rural and urban areas [58,59]. While the results in this section are based on a discussion of long-term extreme temperature exposure in local areas, considering and controlling for the possible influence of long-term adaptability as well as the dependence of local residents and the local industrial structure on climatic conditions, it supplements the results of existing literature, and further emphasizes that the adverse effects of long-term slow local extreme temperature exposure on the rural population are also higher than those in urban areas.

Finally, Panel D of Table 4 illustrates the inequitable effect of extreme temperature on the physical health of the different genders. To sum up, compared with men, women show higher vulnerability in different dimensions of physical health indicators. The negative impact of extreme low-temperature exposure on women’s physical health is especially comprehensive and significant, which is basically consistent with research results in the existing literature. Specifically, extreme cold significantly worsens women’s ADL/IADL, the prevalence of chronic diseases and has a significant negative impact on women’s long-term memory and short-term memory. The damage of extreme cold exposure to women’s cognitive ability is greater than that of men, while the negative impact of extreme cold exposure on men’s ADL/IADL and chronic diseases is not significant. The results of this section further emphasize the vulnerability of women in coping with the health risks of climate change. Compared with men, women’s health is more comprehensively affected by extreme temperature exposure. In addition, the empirical results in the basic regression prove that extreme heat exposure worsens the health of the whole sample of middle-aged and elderly people. The results in Panel D emphasize the gender differences and gender inequalities of extreme temperature exposure. Compared with men, middle-aged and elderly women are sensitive to extreme temperature exposure, especially cold exposures.

## 5. Robustness Checks

In order to verify the consistency of the results in this paper, this section carries out rich robustness tests on index construction, model setting, and identification methods in the basic model and an extended model (see Table 5).

Firstly, considering the possible limitations and subjectivity in the construction of “extreme temperature exposure” indicators, this section uses various alternative measures of extreme temperature exposure indicators to test the research results of the benchmark regression. First, the definition in the existing literature is used to estimate the impact of monthly weather changes on infant birth outcomes [11], and the threshold of extreme temperature is relaxed to 0.7 SD (Panel A of Table 5). Under this loose definition, the regression results are consistent with the main results in the benchmark regression; that is, there is a statistically significant negative impact of extreme temperature on the health status of middle-aged and elderly people in different dimensions.

Secondly, compared with the extreme temperature exposure index based on “daily average temperature” in the basic model (that is, the deviation between “daily average temperature” and historical temperature in the same period is used as the calculation basis), “daily extreme temperature” (that is, daily maximum/minimum temperature) is used as the calculation basis in Panel B of Table 5 and compared with the corresponding local historical temperature in the same period. The corresponding estimation results are also very similar to those in the benchmark model and have a certain robustness.

Thirdly, in order to be consistent with settings on extreme temperature with other literature, Panel C and D of Table 5 redefine “extreme temperature exposure” in the model by using the absolute temperature value. First, based on the most empirical studies, this section defines a daily average absolute temperature above 30 °C and below 0 °C as “extreme high temperature exposure” and “extreme low temperature exposure”, respectively. In Panel C, the days of daily average absolute temperature above 30 °C and below 0 °C in the past year are recalculated as the exposure days of “extreme high temperature” and the exposure days of “extreme low temperature”, and the basic model is then re-estimated. The results of Panel C show that, when the exposure days below 0 °C increase, it has a comprehensive negative impact on the individual’s physical health indicators, including cognitive ability. Exposure days above 30 °C also increase the incidence of chronic diseases in individuals. Then, in accordance with most literature, considering the nonlinear effects of absolute temperature, Panel D also applies the absolute temperature values and divides them into five groups for discussion, i.e., below 0 °C, 0–10 °C, 10–20 °C, 20–30 °C, and above 30 °C. The results show that, compared with the comfortable and moderate temperature zone of 10–20 °C, lower temperature exposure and higher temperature exposure both have adverse effects on an individual’s physical health, and there are differences in the prevalence of chronic diseases, self-reported health level, ADL/IADL, and cognitive ability. However, the above extreme temperature index is based on the definition of absolute temperature, which does not consider the long-term adaptability to the local environment and emphasizes the cold and hot influence of objective temperature. Therefore, it is difficult to directly compare the estimated coefficients with the results of the basic model.

The robustness test results in Table 5 show that, compared with the basic regression, changing the measurement of indicators and model settings does not change the significance and direction of the coefficient, which further verifies the robustness and consistency of the basic regression.

## 6. Discussions and Limitations

The increasing frequency of extreme temperature events in the context of climate change is not only a major environmental issue, but also has implications for public health, agricultural development, fiscal policy, health care, and other aspects. The impacts of extreme temperatures will be widespread and intensive, affecting not only the food, air, water, and housing on which societies depend, across all regions of the world and across income groups. As this study illustrates, it further exacerbates existing inequalities, and affects vulnerable groups within and across countries more frequently and for longer periods of time.

The findings of this paper provide further evidence of the importance of air conditioning and heating devices in developing countries, particularly in mitigating the threats from exposure to extreme temperatures, and further emphasize the importance of such devices in improving the resilience of vulnerable populations to climate change. However, air conditioners and various heating and heating devices also pose serious environmental health risks. As of 2018, air conditioners account for 8.5% of global electricity consumption [6]. It significantly increases global electricity demand and emissions of waste heat, thus further exacerbating the urban heat island effect; on the other hand, the use of air conditioners further leads to significant increases in emissions of carbon dioxide, HFCs, PM2.5, and accelerates ground-level ozone formation, thus contributing to greenhouse gas emission formation, thus exacerbating greenhouse gas emissions and air pollution levels [60,61].

The health risks and threats posed by extreme temperatures require better early warning and protection systems for public health systems. A city-level climate change risk assessment and the early-warning system is needed to provide more health information and health services to the public, especially vulnerable populations. Furthermore, more public-oriented green space systems should be established to ensure equal access to green space for all vulnerable populations [62,63], thereby reducing noise pollution and air pollution. The government should provide residents with more places for social interaction and physical activity, contribute to reducing the urban heat island effect and slow down the process of climate change [64,65], and enhance residents’ adaptation to temperature extremes.

Although the findings of this paper provide a wealth of information and have an important reference value for relevant policies in the future, it must be acknowledged that the study still has the following limitations and there are some elements to be studied in the follow-up.

First, the lack of specific interview dates in the CHARLS database used in this paper (specific visit months are only published for 2015 and the latest release of 2018 data, and visit months for 2011 and 2013 were released in a later update, but no specific visit dates were published for either) makes it impossible to precisely match meteorological data in the studies. Thus, it prevents the discussion and comparison of the immediate impacts and short-, medium-, and long-term effects of extreme temperature exposure on the physical health of middle-aged and older adults. Therefore, the number of days of extreme temperature exposure in the past year in the particular city where the respondent lived was primarily counted. However, in line with the CHARLS tradition, most visits are concentrated in the summer months (the vast majority of visits are in June–August, with only a very small number of special samples requiring additional surveys in other months), which mitigates to some extent the bias that this limitation produces for model estimation. On the other hand, based on the rich physical health indicators and cognitive ability indicators contained in the CHARLS database, this paper constructs several indexes to measure the health status of the middle-aged and elderly populations in a comprehensive manner. However, it must be acknowledged that some of the physical health indicators (including self-rated health status, chronic disease status, and ADL/IADL) are based on the independent responses of middle-aged and elderly respondents, which are still subjective. The accuracy may be affected by their cognitive decline and cross-influence with the cognitive ability measures, thus bringing some measurement error problems. If the subsequent CHARLS database can disclose more abundant and accurate interview dates and publish the indicators of blood test information collected in each year’s survey (at present, only some years are disclosed, and the response rate of blood test data is only about 60%, which may have certain sample selection problems), it can use more objective blood test indicators (e.g., blood glucose, blood pressure, several blood lipid indicators, etc.) to more realistically reflect the physical health status of the respondents; and combined with accurate meteorological data, thus better realizing the causal identification process in this study and improving the accuracy and robustness of the estimates in this paper.

Secondly, this paper is based on the definition of “local relative temperature extremes”, which defines the degree of deviation of the current daily average temperature from the local historical monthly average temperature as the presence of extreme temperature exposure. Thus, the key identifying assumption of this empirical strategy is that temperature changes are independent of other unobserved determinants of health for a given individual under continuous changes in physical health variables. However, it has to be admitted that even based on such indicator construction and panel model setting, it is still impossible to fully control for all other variables that are related to local changes in temperature and may directly affect the physical health status of individuals, and the estimation bias caused by omitted variables may still have an impact on the estimation results. A more accurate and long-term meteorological database and a micro-database with a larger sample size are needed to further complement and validate the results of this paper by finding instrumental variables or more exogenous shocks to complete the causal inference.

Finally, the findings provide new evidence on the long-term accumulative costs of temperature extremes on human capital in middle-aged and elderly populations, contributing to the understanding of the social costs of climate change and the associated health inequalities. However, it must be acknowledged that, limited by the availability of data, this paper only attempts to analyze the effect of having adaptive tools such as air conditioning on mitigating the negative effects of temperature extremes using the micro-individual level, and still lacks an in-depth analysis and validation of its impact mechanisms. The complex mechanisms underlying the effects of localized relative temperature extremes on chronic diseases, impairments in daily activities, cognitive performance, depression, cognitive processes, and other emotional and behavioral aspects are still under investigation, and further research is needed to more fully understand the health risks associated with climate change.

## 7. Conclusions

In recent decades, various studies have proved that climate change will pose great challenges and threats to population health, and the adverse effects on physical health are also one of the outcome variables to which scholars pay the most attention. Especially with the acceleration of the aging process in China, the elderly has gradually become one of the most vulnerable groups that are negatively affected.

Based on large-scale household survey data and national meteorological historical data, this paper considers the self-reported health level, daily activity disorder (ADL/IADL), chronic diseases, and cognitive ability of middle-aged and elderly people as proxy variables of individual health, describes the physical health status of middle-aged and elderly people in China in a multi-dimensional way, and analyzes the impact of long-term cumulative exposure to extreme temperatures on the physical health of middle-aged and elderly people in China.

In order to capture unobservable regional and population heterogeneity and obtain consistent estimation results, this paper employs CHARLS three-year unbalanced panel data to estimate the fixed effect model. The results show that long-term exposure to extreme heat and cold will significantly affect the health of individuals. Considering the average annual level of heat exposure, as one extra heat exposure day, the self-reported health status of individuals decreases by 3.22%, and the ADL/IADL and chronic diseases increase significantly by 1.19% and 1.30%, respectively, which leads to the deterioration of individual health status. Extreme cold exposure can similarly damage physical health, but its impact on self-reported health status and the number of chronic diseases is much smaller than that of extreme heat. Similarly, from the perspective of cognitive ability, extreme temperature exposure also has obvious negative effects. In addition, comparatively speaking, the negative effects of extreme heat exposure are comprehensive and significant in both summer and winter. At the same time, the important role of air conditioning and heating equipment in helping people adapt to climate change requires more attention. Air conditioning and heating equipment alleviate the impact of extreme temperature exposure on individual health to a great extent and play an effective role in helping people adapt to environmental temperature changes. Further sub-sample results show that the elderly, women, and rural residents are vulnerable groups, and they are at a disadvantage in coping with the impact of extreme temperature exposure on various health indicators. In particular, women are more vulnerable to the negative effects of extreme low temperatures. In order to verify the robustness and consistency of the model results, this paper uses a variety of measurements of extreme temperature, and all of them led to results in line with expectations.

This paper further emphasizes the health threats and challenges of extreme temperatures, which can help the public to better understand the impact of climate change, improve the ability of vulnerable people to cope with and adapt to climate change, and better realize health equity and environmental justice. As an important developing country in the world, China has taken strong measures to reduce greenhouse gas emissions and slow down climate change in recent years and has achieved certain results. However, China’s carbon intensity is still very high. Using China’s data to study the relationship between extreme temperature events and population health against the background of climate change is not only of great significance to China’s climate change policy and the health of the population, but will also lead to global economic and technological reforms and have great significance for protecting the ecology and the promoting of international cooperation on climate change.

## Figures and Tables

**Table 1:** Descriptive statistics.

Variables	Observations	Mean	S.D.	Min	Max
Panel A: Physical health variables					
Self-reported health status	50,059	3.14	0.99	1.00	5.00
ADL/IADL	50,059	0.27	0.45	0.00	1.00
Suffer from chronic diseases	50,059	1.19	1.33	0.00	10.00
Cognitive Ability					
Short-term memory	50,059	3.58	2.11	0.00	10.00
Long-term memory	50,059	2.85	2.02	0.00	10.00
Mathematics test	50,059	2.63	2.05	0.00	5.00
Panel B: Climate Variables					
Z value	50,059	0.26	1.05	−4.60	7.08
Hot exposure days	50,059	100.77	41.91	4.00	357.00
Cold exposure days	50,059	77.53	39.48	0.00	296.00
Hot exposure days in summer	50,059	27.72	14.53	0.00	92.00
Cold exposure days in winter	50,059	20.23	11.01	0.00	73.00
Panel C: Control Variables					
Precipitation	50,059	2.74	1.52	0.22	6.96
Wind speed	50,059	2.12	0.74	0.84	4.96
Duration of sunshine	50,059	5.19	1.44	2.13	8.60
Relative humidity	50,059	68.59	8.86	42.94	85.01
Air pressure	50,059	970.37	62.72	741.89	1016.78
PM2.5	50,059	37.43	16.81	2.41	74.81
Age	50,059	59.93	10.02	20.00	101.00
Age^2^	50,059	3691.95	1250.67	400.00	10,201.00
Education level	50,059	2.76	1.37	1.00	5.00
Receive medical	50,059	0.76	0.43	0.00	1.00
Number of children	50,059	2.73	1.45	0.00	11.00
Number of sons	50,059	1.54	1.02	0.00	8.00
PCE (Personal Consumption Expenditure, Excluding Medical Expenditure, Yuan)	50,059	4.23	4.42	0.00	12.93

Data source: CHARLS 2011, 2013, 2015 Survey Data.

**Table 2:** Results of the basic model.

Variables	(1)	(2)	(3)	(4)	(5)	(6)
	Self-Reported Health	ADL/IADL	Chronic Disease	Short-Term Memory	Long-Term Memory	Mathematics Test
Heat Exposure Days	−0.0019 ***	0.0003 *	0.0019 ***	−0.0008	−0.0017 **	−0.0017 **
	(0.000)	(0.000)	(0.000)	(0.001)	(0.001)	(0.001)
Cold Exposure Days	−0.0011 ***	−0.0004	0.0007 **	−0.0031 ***	−0.0030 ***	0.0011
	(0.000)	(0.000)	(0.000)	(0.001)	(0.001)	(0.001)
Precipitation	−0.0411 ***	0.0012	−0.0096	−0.0123	−0.0297	−0.0283
	(0.005)	(0.005)	(0.007)	(0.021)	(0.021)	(0.021)
Wind Speed	−0.0373 ***	0.0270 **	0.0508 ***	0.1931 ***	0.1122 **	−0.1602 ***
	(0.008)	(0.011)	(0.015)	(0.049)	(0.047)	(0.045)
Sunshine Duration	−0.0247 ***	0.0050	0.0104	0.0199	0.0071	0.0466*
	(0.006)	(0.006)	(0.009)	(0.025)	(0.025)	(0.025)
Relative Humidity	0.0069 ***	0.0046 ***	0.0034 **	0.0136 ***	0.0101 **	0.0001
	(0.001)	(0.001)	(0.001)	(0.004)	(0.004)	(0.004)
Air Pressure	−0.0008 ***	0.0002	0.0126 ***	0.0059	−0.0054	0.0011
	(0.000)	(0.002)	(0.003)	(0.008)	(0.008)	(0.008)
PM2.5	−0.0030 ***	0.0019 **	0.0057 ***	0.0003	−0.0033	−0.0069 **
	(0.000)	(0.001)	(0.001)	(0.003)	(0.003)	(0.003)
Age	0.0323 ***	−0.0084 *	0.0052	0.1657 ***	0.1503 ***	0.0950 ***
	(0.005)	(0.005)	(0.006)	(0.032)	(0.024)	(0.020)
Age^2^	−0.0002 ***	0.0001 ***	−0.0001	−0.0014 ***	−0.0013 ***	−0.0009 ***
	(0.000)	(0.000)	(0.000)	(0.000)	(0.000)	(0.000)
Education level (reference group: never attended school)						
Not finish elementary school	−0.0073	0.0600 **	0.0074	0.2014 *	0.1205	0.0859
	(0.017)	(0.028)	(0.046)	(0.108)	(0.099)	(0.105)
Elementary school	−0.0816 ***	0.0083	−0.0729	0.3507 **	0.3379 ***	0.2530 *
	(0.016)	(0.032)	(0.053)	(0.138)	(0.126)	(0.130)
Middle school	−0.1592 ***	0.0105	−0.0827	0.3747 **	0.2012	−0.0323
	(0.017)	(0.040)	(0.060)	(0.171)	(0.163)	(0.167)
High school and above	−0.2911 ***	0.0179	0.0033	0.2043	0.0075	0.0971
	(0.019)	(0.048)	(0.094)	(0.256)	(0.256)	(0.252)
Receive Medical	−0.3608 ***	−0.0282 ***	−0.0519 ***	0.0494 **	0.1299 ***	−0.0616 **
	(0.009)	(0.006)	(0.009)	(0.025)	(0.024)	(0.025)
Number of children	0.0246 ***	0.0034	−0.0051	0.0125	0.0242	−0.0100
	(0.005)	(0.005)	(0.009)	(0.023)	(0.021)	(0.021)
Number of sons	−0.0062	0.0002	−0.0024	−0.0663 **	−0.0569 **	−0.0313
	(0.006)	(0.006)	(0.008)	(0.029)	(0.027)	(0.026)
ln(PCE)	−0.0053 ***	−0.0005	0.0160 ***	−0.0050	−0.0031	0.0117 ***
	(0.001)	(0.001)	(0.001)	(0.003)	(0.003)	(0.003)
Constants	2.6488 ***	−0.2828	−11.9096 ***	−8.0731	3.3999	−0.4130
	(0.217)	(1.774)	(2.777)	(7.774)	(7.621)	(7.721)
Observation	48,434	50,025	50,059	50,059	50,059	50,059

Note: (1) Data source: CHARLS survey data in 2011, 2013, and 2015. (2) All standard errors are robust standard errors. (3) All models include all control variables and individual, year, and city fixed effects. (4) All models are fixed effect models. (5) ***, ** and * represent significant levels of 1%, 5% and 10%, respectively.

**Table 3:** Regression results of the adaptations behaviors.

Variables	(1)	(2)	(3)	(4)	(5)	(6)
	Self-Reported Health	ADL/IADL	Chronic Disease	Short-Term Memory	Long-Term Memory	Mathematics Test
Panel A: Extreme temperature in summer						
Heat Exposure Days	−0.0041 ***	0.0012 ***	0.0030 ***	0.0019	0.0021	−0.0006
	(0.000)	(0.000)	(0.001)	(0.002)	(0.002)	(0.002)
Cold Exposure Days	−0.0032 ***	−0.0006	0.0029 ***	0.0031	0.0049 **	0.0022
	(0.001)	(0.000)	(0.001)	(0.002)	(0.002)	(0.002)
Constants	2.7202 ***	−0.9160	−11.2337 ***	−7.5447	3.5991	−1.2911
	(0.222)	(1.799)	(2.801)	(7.826)	(7.675)	(7.801)
Observation	48,434	50,025	50,059	50,059	50,059	50,059
Panel B: Extreme temperature in winter						
Heat Exposure Days	−0.0051 ***	0.0004	0.0036 ***	−0.0042 ***	−0.0060 ***	−0.0007
	(0.000)	(0.000)	(0.000)	(0.001)	(0.001)	(0.001)
Cold Exposure Days	−0.0008	−0.0004	0.0003	−0.0009	−0.0021	0.0010
	(0.001)	(0.000)	(0.001)	(0.002)	(0.002)	(0.002)
Constants	2.8398 ***	−0.3755	−12.3979 ***	−8.2553	2.8685	0.6988
	(0.208)	(1.794)	(2.813)	(7.835)	(7.686)	(7.804)
Observation	48,434	50,025	50,059	50,059	50,059	50,059
Panel C: No air conditioning in summer						
Heat Exposure Days	−0.0017 ***	0.0020 ***	0.0027 ***	−0.0017	−0.0013	−0.0035 *
	(0.001)	(0.001)	(0.001)	(0.002)	(0.002)	(0.002)
Constants	2.8309 ***	−1.0320	−14.9659 ***	−11.6212	0.2262	0.9602
	(0.245)	(2.195)	(3.443)	(9.253)	(8.831)	(9.067)
Observation	34,820	36,010	36,036	36,036	36,036	36,036
With air conditioning in summer						
Heat Exposure Days	−0.0032	−0.0000	0.0058	0.0087 **	0.0122 ***	0.0091 **
	(0.018)	(0.002)	(0.021)	(0.004)	(0.004)	(0.004)
Constants	2.9660 ***	9.0672	−2.2749	47.9498 **	49.0231 **	−1.9222
	(0.577)	(11.102)	(6.542)	(21.125)	(22.549)	(21.821)
Observation	13,614	14,015	14,023	14,023	14,023	14,023
Panel D: No heating equipment in winter						
Cold Exposure Days	−0.0036 ***	0.0009	0.0030 ***	−0.0084 **	−0.0039	−0.0064 **
	(0.001)	(0.003)	(0.001)	(0.003)	(0.003)	(0.003)
Constants	2.7266 ***	0.0604	−11.9312 **	50.1098 **	40.9449 **	39.2510 **
	(0.256)	(16.821)	(5.061)	(18.330)	(17.430)	(17.811)
Observation	17,496	18,379	18,396	18,396	18,396	18,396
With heating equipment in winter						
Cold Exposure Days	−0.0003	−0.0041	−0.0026	−0.0053	0.0011	0.0092
	(0.001)	(0.005)	(0.002)	(0.007)	(0.006)	(0.007)
Constants	3.1290 ***	−33.7426 *	−11.8325	−41.2955	−27.6753	27.0247
	(0.335)	(17.850)	(13.029)	(29.351)	(28.741)	(31.271)
Observation	30,904	31,611	31,628	31,628	31,628	31,628

Note: (1) Data source: CHARLS survey data in 2011, 2013 and 2015. (2) All standard errors are robust standard errors. (3) All models include all control variables and individual, year, and city fixed effects. (4) All models are fixed effect models. (5) All models contain a complete set of control variables, namely precipitation, wind speed, sunshine duration, relative humidity, air pressure, PM2.5, age (including square term), education level, medical insurance, number of children, number of sons, and PCE (logarithm of personal consumption expenditure, excluding medical expenditure, Yuan). (6) ***, ** and * represent significant levels of 1%, 5%, and 10%, respectively.

**Table 4:** Regression results of the sub-samples.

Variable	(1)	(2)	(3)	(4)	(5)	(6)	(7)	(8)	(9)	(10)	(11)	(12)
	Self-Reported Health		ADL/IADL		Chronic Diseases		Short-Term Memory		Long-Term Memory		Mathematics Test	
Panel A: Age	<60	>60	<60	>60	<60	>60	<60	>60	<60	>60	<60	>60
Heat Exposure Days	−0.0014 ***	−0.0020 ***	0.0006	0.0017	0.0012 ***	0.0024 ***	0.0007	−0.0006	−0.0009	−0.0014	−0.0008	−0.0015
	(0.000)	(0.000)	(0.001)	(0.001)	(0.000)	(0.000)	(0.001)	(0.001)	(0.001)	(0.001)	(0.001)	(0.001)
Cold Exposure Days	−0.0010 ***	−0.0009 ***	0.0006	0.0039 ***	0.0004	0.0005	−0.0015	−0.0022	−0.0018	−0.0018	0.0008	−0.0031 **
	(0.000)	(0.000)	(0.001)	(0.002)	(0.000)	(0.001)	(0.001)	(0.001)	(0.001)	(0.001)	(0.001)	(0.001)
Observation	27,216	21,218	27,970	22,055	27,988	22,071	27,988	22,071	27,988	22,071	27,988	22,071
Panel B: Regional	North	South	North	South	North	South	North	South	North	South	North	South
Heat Exposure Days	−0.0003	−0.0020 ***	0.0039 ***	0.0008	0.0014 ***	0.0017 ***	0.0001	−0.0034 ***	−0.0009	−0.0042 ***	−0.0027 **	−0.0007
	(0.000)	(0.000)	(0.001)	(0.001)	(0.000)	(0.000)	(0.001)	(0.001)	(0.001)	(0.001)	(0.001)	(0.001)
Cold Exposure Days	−0.0005	−0.0007 ***	0.0039 ***	0.0005	−0.0001	0.0014 ***	−0.0025 *	−0.0038 ***	−0.0026 **	−0.0036 ***	0.0004	0.0009
	(0.000)	(0.000)	(0.001)	(0.001)	(0.000)	(0.000)	(0.001)	(0.001)	(0.001)	(0.001)	(0.001)	(0.001)
Observation	22,893	25,541	23,684	26,341	23,704	26,355	23,704	26,355	23,704	26,355	23,704	26,355
Panel C: Urban	Urban	Rural	Urban	Rural	Urban	Rural	Urban	Rural	Urban	Rural	Urban	Rural
Heat Exposure Days	−0.0016 ***	−0.0012 ***	0.0009	0.0023 ***	0.0013 ***	0.0020 ***	0.0036 ***	−0.0029 ***	0.0021	−0.0038 ***	−0.0004	−0.0019 **
	(0.000)	(0.000)	(0.001)	(0.001)	(0.000)	(0.000)	(0.001)	(0.001)	(0.001)	(0.001)	(0.001)	(0.001)
Cold Exposure Days	−0.0010 ***	−0.0006 **	0.0020 *	0.0009	0.0007	0.0007 *	−0.0021	−0.0040 ***	−0.0019	−0.0044 ***	0.0026 *	−0.0002
	(0.000)	(0.000)	(0.001)	(0.001)	(0.001)	(0.000)	(0.001)	(0.001)	(0.001)	(0.001)	(0.001)	(0.001)
Observation	19,302	29,132	20,075	29,950	20,096	29,963	20,096	29,963	20,096	29,963	20,096	29,963
Panel D: Gender	Male	Female	Male	Female	Male	Female	Male	Female	Male	Female	Male	Female
Heat Exposure Days	−0.0017 ***	−0.0016 ***	0.0012	0.0013	0.0014 ***	0.0020 ***	−0.0007	−0.0003	−0.0016	−0.0016	0.0012	0.0020 **
	(0.000)	(0.000)	(0.001)	(0.001)	(0.000)	(0.000)	(0.001)	(0.001)	(0.001)	(0.001)	(0.001)	(0.001)
Cold Exposure Days	−0.0010 ***	−0.0007 ***	0.0011	0.0020 *	0.0002	0.0009 **	0.0029 **	0.0037 ***	0.0029 **	0.0036 ***	0.0011	0.0008
	(0.000)	(0.000)	(0.001)	(0.001)	(0.000)	(0.000)	(0.001)	(0.001)	(0.001)	(0.001)	(0.001)	(0.001)
Observation	23,064	25,366	23,850	26,170	23,871	26,183	23,871	26,183	23,871	26,183	23,871	26,183

Note: (1) Data source: CHARLS survey data in 2011, 2013, and 2015. (2) All standard errors are robust standard errors. (3) All models include all control variables and individual, year, and city fixed effects. (4) All models are fixed effect models. (5) All models contain a complete set of control variables, namely precipitation, wind speed, sunshine duration, relative humidity, air pressure, PM2.5, age (including square term), education level, medical insurance, number of children, number of sons and PCE (logarithm of personal consumption expenditure, excluding medical expenditure). (6) ***, ** and * represent significant levels of 1%, 5%, and 10%, respectively.

**Table 5:** Robustness test results.

Variables	(1)	(2)	(3)	(4)	(5)	(6)
	Self-Reported Health	ADL/IADL	Chronic Disease	Short-Term Memory	Long-Term Memory	Mathematics Test
Panel A: Relative Indicate − 0.7 SD						
Heat Exposure Days	−0.0020 ***	0.0030 ***	0.0019 ***	−0.0006	−0.0019 *	−0.0028 ***
	(0.000)	(0.001)	(0.000)	(0.001)	(0.001)	(0.001)
Cold Exposure Days	−0.0013 ***	0.0033 ***	0.0007 *	−0.0018 *	−0.0023 **	−0.0011
	(0.000)	(0.001)	(0.000)	(0.001)	(0.001)	(0.001)
Constants	3.0601 ***	−12.8927 **	−11.7080 ***	−8.9354	3.0885	0.9777
	(0.255)	(6.340)	(2.801)	(7.783)	(7.650)	(7.790)
Observation	48,434	50,025	50,059	50,059	50,059	50,059
Panel B: Relative index daily extreme temperature − 1.96 SD						
Heat Exposure Days	−0.0015 ***	0.0009 *	0.0011 ***	0.0008	0.0013 **	−0.0008
	(0.000)	(0.001)	(0.000)	(0.001)	(0.001)	(0.001)
Cold Exposure Days	−0.0004 ***	0.0005	0.0000	−0.0013 *	0.0008	0.0010
	(0.000)	(0.001)	(0.000)	(0.001)	(0.001)	(0.001)
Constants	3.2047 ***	−9.7924	−11.4689 ***	−10.1446	5.8757	2.0179
	(0.216)	(6.559)	(2.874)	(7.989)	(7.845)	(7.943)
Observation	48,434	50,025	50,059	50,059	50,059	50,059
Panel C: Absolute temperature index						
Daily temperature > 30 days	−0.0005	0.0010	0.0043 ***	0.0025	0.0003	−0.0016
	(0.001)	(0.002)	(0.001)	(0.002)	(0.002)	(0.002)
Constants	3.4732 ***	−10.4767	−9.8489 ***	−6.7422	4.1908	−0.4472
	(0.213)	(6.386)	(2.810)	(7.865)	(7.727)	(7.852)
Observation	48,265	49,880	49,880	49,880	49,880	49,880
Daily temperature < 0 days	−0.0008 ***	0.0039 ***	0.0022 ***	−0.0046 ***	−0.0037 ***	−0.0003
	(0.000)	(0.001)	(0.000)	(0.001)	(0.001)	(0.001)
Constants	3.4919 ***	−12.9872 **	−12.8080 ***	−10.2434	2.0678	0.0587
	(0.208)	(6.456)	(2.817)	(7.852)	(7.724)	(7.857)
Observation	48,434	50,025	50,059	50,059	50,059	50,059
Panel D: Absolute temperature index						
Daily temperature < 0 days	−0.0000	0.0046 ***	0.0026 ***	−0.0046 ***	−0.0029 *	0.0018
	(0.000)	(0.001)	(0.001)	(0.002)	(0.002)	(0.002)
0 < daily temperature ≤ 10	−0.0013 ***	0.0007	0.0009 **	0.0001	0.0010	0.0025 **
	(0.000)	(0.001)	(0.000)	(0.001)	(0.001)	(0.001)
20 < daily temperature ≤ 30	−0.0014 ***	0.0002	0.0012 **	0.0021	0.0005	0.0002
	(0.000)	(0.001)	(0.000)	(0.001)	(0.001)	(0.001)
Daily temperature > 30 days	−0.0007	0.0002	0.0055 ***	0.0042	−0.0002	−0.0017
	(0.001)	(0.002)	(0.001)	(0.003)	(0.003)	(0.003)
Constants	3.4790 ***	−13.2390 **	−10.7925 ***	−8.6735	2.3206	0.2959
	(0.226)	(6.497)	(2.829)	(7.938)	(7.797)	(7.957)
Observation	48,434	50,025	50,059	50,059	50,059	50,059

Note: (1) Data source: CHARLS survey data in 2011, 2013, and 2015. (2) All standard errors are robust standard errors. (3) All models include all control variables and individual, year, and city fixed effects. (4) All models are fixed effect models. (5) All models contain a complete set of control variables, namely precipitation, wind speed, sunshine duration, relative humidity, air pressure, PM2.5, age (including square term), education level, medical insurance, number of children, number of sons and PCE (logarithm of personal consumption expenditure, excluding medical expenditure). (6) ***, ** and * represent significant levels of 1%, 5%, and 10%, respectively.

## Data Availability

The climate-related data can be obtained from Climate Data Online (CDO) of the U.S. National Oceanic and Atmospheric Administration (NOAA), please refer to: https://www.ncdc.noaa.gov/cdo-web/, accessed on 19 November 2022. And the population data can be obtained from China Health and Retirement Longitudinal Study (CHARLS) program by the China Social Science Research Center of Peking University, please refer to the official website: http://charls.pku.edu.cn/, accessed on 19 November 2022.
